# Messengers from the deep: Fossil wadsleyite-chromite microstructures from the Mantle Transition Zone

**DOI:** 10.1038/srep16484

**Published:** 2015-11-13

**Authors:** Takako Satsukawa, William L. Griffin, Sandra Piazolo, Suzanne Y. O’Reilly

**Affiliations:** 1 Australian Research Council Centre of Excellence for Core to Crust Fluid Systems/GEMOC, Department of Earth and Planetary Sciences, Macquarie University, Sydney, NSW 2109, Australia

## Abstract

Investigations of the Mantle Transition Zone (MTZ; 410–660 km deep) by deformation experiments and geophysical methods suggest that the MTZ has distinct rheological properties, but their exact cause is still unclear due to the lack of natural samples. Here we present the first direct evidence for crystal-plastic deformation by dislocation creep in the MTZ using a chromitite from the Luobusa peridotite (E. Tibet). Chromite grains show exsolution of diopside and SiO_2_, suggesting previous equilibration in the MTZ. Electron backscattered diffraction (EBSD) analysis reveals that olivine grains co-existing with exsolved phases inside chromite grains and occurring on chromite grain boundaries have a single pronounced crystallographic preferred orientation (CPO). This suggests that olivine preserves the CPO of a high-pressure polymorph (wadsleyite) before the high-pressure polymorph of chromite began to invert and exsolve. Chromite also shows a significant CPO. Thus, the fine-grained high-pressure phases were deformed by dislocation creep in the MTZ. Grain growth in inverted chromite produced an equilibrated microstructure during exhumation to the surface, masking at first sight its MTZ deformation history. These unique observations provide a window into the deep Earth, and constraints for interpreting geophysical signals and their geodynamic implications in a geologically robust context.

Seismological studies of Earth’s mantle reveal three distinct changes in seismic velocity, at depths of 410, 660 and 2700 km; the interval between 410 and 660 km is termed the Mantle Transition Zone (MTZ). The first two changes are explained by phase transitions in olivine, the dominant mineral of the upper mantle; olivine to wadsleyite at 410 km, and ringwoodite to Mg-silicate perovskite (bridgmanite) plus ferropericlase at 660 km. Tomographic images of cold slabs in the lower mantle, and the displacements of the 410-km and 660-km discontinuities beneath subduction zones imply whole-mantle convection^1^. Currently, our knowledge of the rheology of the deep mantle and MTZ is based largely on experimental studies of forsterite, wadsleyite and ringwoodite^1^,^2^. Actual samples of mantle rocks from deeper than 200 km are rare, and we still know little about the rheological properties and deformation behavior of olivine polymorphs in the lower parts of the upper mantle.

Luobusa is one of several large peridotite massifs along the Yarlung-Zangbo suture zone of southern Tibet, which marks the boundary between the Indian and Asian blocks^3^. The podiform chromitites in the Luobusa peridotite have received much attention because they include many ultra-high-pressure (UHP) phases, such as microdiamonds (found in mineral separates and *in situ*)^4^, Si-rutile and coesite, as well as a range of highly reduced native elements, carbides and nitrides^4^,^5^,^6^.

Recently, a basic model for the subduction, MTZ UHP metamorphism and exhumation of the Tibetan peridotites has been presented^7^. The presence of a cubic Mg-silicate with inverse-spinel structure in the same ore body^8^ further supports MTZ depths for these rocks. These observations indicate that Luobusa chromitites have experienced much higher pressures than the more common ophiolitic chromitites, whose history is restricted to the uppermost part of the mantle^9^.

The chromites commonly show exsolution of diopside, enstatite and coesite^4^,^10^, suggesting inversion from a high-pressure polymorph that incorporated Ca and Si, such as the Ca-Ferrite (CF; CaFe_2_O_4_) structure (>15 GPa at TZ temperatures of 1400–1500 °C)^8^,^10^,^11^. Several recent experimental studies show variations in the details of the phase diagram for chromite under MTZ conditions. Ishii *et al.* (2014, 2015)^12^,^13^ suggested that chromite breaks down to an intermediate mLD-type (Mg, Fe)_2_Cr_2_O_5_ + Cr_2_O_3_ eskolaite assemblage (12–15 GPa) before the CF structure is formed. However, these experiments may not be relevant to real chromite, which contains significant levels of aluminum. Multianvil experiments using natural Al-rich Luobusa chromitite + diopside or SiO_2_ as a starting materials (14 GPa, 1600 °C)^14^,^15^,^16^ found that chromite starts to transform into (Fe, Mg)_2_(Al, Cr)_2_O_5_ with significant Ca or Si substitution on the Al site. Although further experiments are required, the intermediate phase is orthorhombic in all cases (as is the CF-type). Here we refer to the group of suggested high-pressure chromite phases as HPP (High Pressure Polymorph)-chromite.

The apparent deep recycling of some podiform chromitites (and their host peridotites) implies previously unrecognized types of mantle dynamics, and requires reinterpretation of the history of the lithospheric mantle in some ophiolites. This contribution presents an electron backscattered diffraction (EBSD) study of UHP massive chromitites from the Luobusa peridotite (E. Tibet, China), shedding light on the deformation behavior of high-pressure phases at MTZ conditions. We provide the first direct evidence of crystal-plastic deformation, evidenced by mineral CPOs and substructures interpreted to have formed by dislocation creep in the UHP phases wadsleyite (now olivine) and HPP-chromite, now normal spinel-structured chromite.

## Results

**Figure 1:**
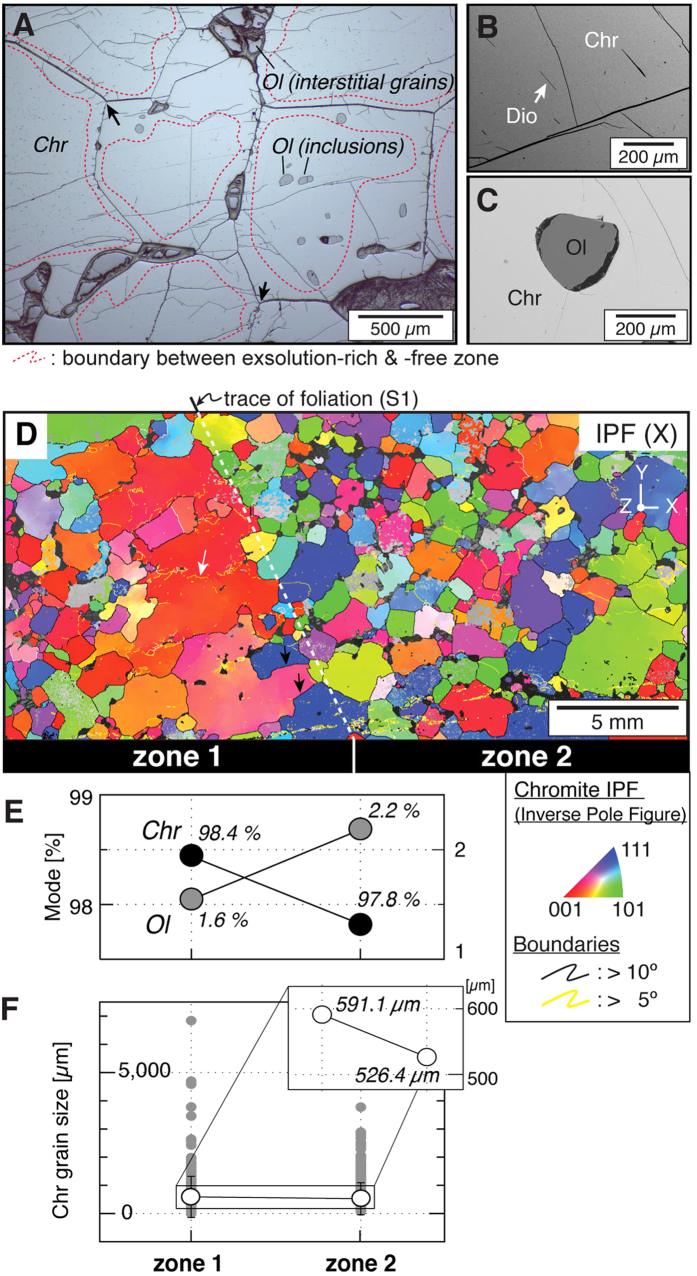
Microstructure of Luobusa chromitite. (**A–C**) Back-scattered electron (BSE) images of Luobusa chromitite. (**A**) Equilibrated microstructure of chromite with 120° triple junctions (black arrow) and smooth, nearly straight grain boundaries. Note the dendritic, bifurcating cracks radiating normal to the grain boundaries. Olivine occurs as inclusions and along grain boundaries. Interstitial grains are partially or completely serpentinized. (**B**) Exsolved diopside needles (Dio) in chromite (Chr). (**C**) Olivine inclusion (Ol) in chromite (Chr). (**D**) Colour coded EBSD map showing crystal orientation changes relative to the X direction of the sample reference frame. White and black arrows represent subgrain boundary and migrated grain boundary, respectively. Modal composition (**E**), and mean grain size and grain size distribution of chromites (**F**) of zone 1 and 2 in (**D**).

**Figure 2:**
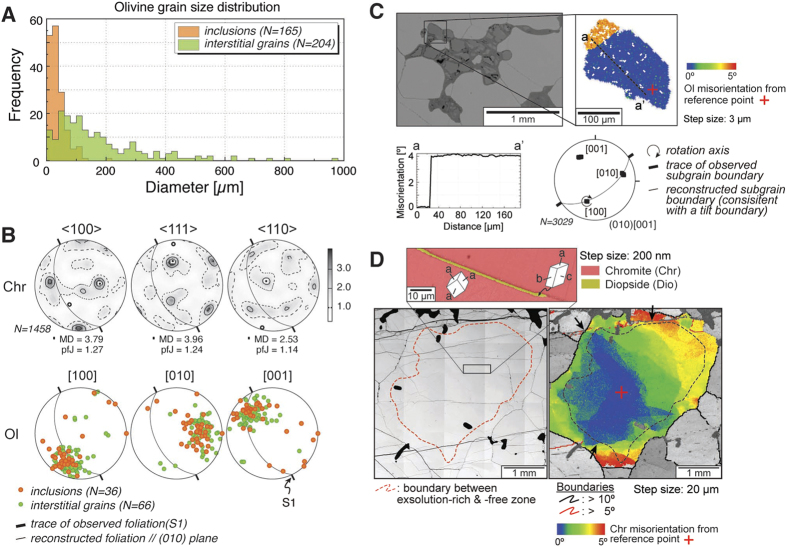
Microstructural characteristics of chromite, olivine and diopside. (**A**) Grain-size distribution of olivine inclusions and interstitial grains. (**B**) CPO of chromites (Chr) and olivines (Ol). Lower hemisphere, equal-area stereographic projections. MD: maximum density, pfJ: index of fabric intensity^59^. (**C**) Representative subgrain-boundary analysis of interstitial olivine. BSE image of interstitial olivines in chromite shows that they look like small grains, but the apparent small size is due to serpentinization. Cumulative orientation map and misorientation profile along the dotted black line in EBSD map showing misorientation from reference orientation (red cross). Olivine CPOs in EBSD map shows rotation axis is [100] and slip system is (010)[001]. (**D**) BSE image and EBSD map of chromite with exsolved diopside needles. EBSD map showing change of orientation from reference orientation (red cross). Although few subgrain boundaries and misorientation in the rim can be recognized, they are related to later fracturing during decompression (black arrows).

The massive chromitite studied here is similar to previously-reported samples^10^ (Mg# = 71; Table S1); it has an apparently equilibrated, near-equigranular microstructure (Fig. 1a) with a mean grain diameter of 580 μm. Dendritic cracks radiate from straight grain boundaries, consistent with expansion of chromite grains during decompression. Needles of exsolved diopside (Figs 1b, S1) are abundant in the cores of chromite grains, but absent in the rims (Fig. 1a). Olivine (Fo_98_; Table S1) occurs both as inclusions in chromite and interstitially along grain boundaries (Fig. 1a,c). In general, inclusions are much smaller (range: 1.7–205 μm; average 37 ± 30 μm) than interstitial grains (range: 4.3–979 μm; average 179.0 ± 161.3 μm) (Figs 1a and 2a).

The sample consists of two zones: Zone 1 contains larger chromite grains (591vm *vs* 526 μm) with slightly higher median aspect ratios (1.73 *vs* 1.70) and lower abundance of olivine (1.6 area% *vs* 2.2 area%) than zone 2 (Fig. 1e,f). The line separating these two zones is inferred to represent the trace of the foliation in this rock (Fig. 1d). Chromite subgrain and grain boundaries are commonly oriented perpendicular to foliation (S1) (white arrow in Fig. 1d), thus the longest grain axis of chromite grains is subhorizontal. The grain elongation perpendicular to foliation is interpreted to reflect late subgrain boundary evolution to form grain boundaries by recovery (black arrow in Fig. 1d).

EBSD analysis shows that the chromite grains have a significant CPO (Fig. 2b), despite their equilibrated near-equigranular microstructure with 120° triple junctions and smooth, nearly straight grain boundaries (Fig. 1a), and relatively low mean internal misorientation per grain (range: 0.06–7.41; average: 0.66 ± 0.82). Well-developed subgrain boundaries are rare although some distinct crystal bending is observed (Figs 1d, S1). EBSD mapping reveals that crystal-plastic deformation has resulted in continuous crystal bending and formation of well-developed subgrain boundaries in the interstitial olivines (Figs 2c, S2). In these grains, these subgrain boundaries allow determination of the activated slip system utilizing the orientation of the misorientation axis, as defined by the lattice dispersion paths, and orientations of the subgrain boundary trace^17^,^18^,^19^,^20^,^21^,^22^,^23^,^24^,^25^,^26^,^27^,^28^,^29^. The small size of the olivine inclusions precludes quantitative substructure analysis.

A well-defined low-angle subgrain boundary (2–10° orientation change across the boundary) is shown in Figs 2(c) and S2. The trace of this boundary does not lie in a plane perpendicular to the misorientation axis as determined by the dispersion paths depicted in the pole figure. Thus, a twist-boundary model can be eliminated for this subgrain boundary. A steep plane that contains the subgrain-boundary trace and the determined misorientation axis [100] is the probable subgrain boundary plane, in this case (001) comprising an array of edge dislocations lying in (001). Hence, subgrain-boundary characteristics are consistent with the activation of the (010)[001] slip system. Olivine (both inclusions and interstitial grains) also shows the same, pronounced CPO with the (010) plane parallel to foliation, consistent with the activation of (010)[001] slip.

The small olivine inclusions in chromite commonly occur in areas where the chromite host contains exsolved needles of diopside (Figs 1a, 2d, S1). All diopside needles show a uniform crystallographic orientation relative to the host chromite, suggesting that they exsolved from a HPP-chromite in a topotaxial relationship with the host: [100]_dio_//<111>_chr_, [010] _dio_//<110> _chr_, and [001] _dio_//<112> _chr_ (Figs 2d, S3). No significant crystal bending of diopside needles was detected, however it should be noted that the needles are too thin to allow unequivocal quantification of substructures.

## Significance and Discussion

The abundance of exsolved diopside needles in the cores of chromite grains, *vs* their absence in the rims (Fig. 1a), indicates that grain growth occurred after the transition from the orthorhombic HPP-chromite to cubic chromite. Grain growth occurs by grain boundary migration between pre-existing grains, allowing readjustment of mineral compositions in the “swept” area to one in equilibrium in the prevailing PT conditions, while maintaining crystallographic near-continuity with the core^29^,^30^,^31^,^32^,^33^. The grain-size difference between the enclosed and interstitial olivine grains is consistent with the evidence for grain boundary migration, because grain boundaries can readily migrate across small grains but can be pinned by larger grains^34^,^35^,^36^,^37^,^38^,^39^. A decrease in the rate of grain growth due to the presence of grains of a second phase also is consistent with the finer grain size and higher abundance of olivine in zone 2 relative to zone 1 (Fig. 1e).

While deformation features such as continuous lattice bending and subgrains perpendicular to foliation are present in the chromite, the chromite CPO patterns do not resemble typical deformation- or growth-induced textures known from cubic phases^40^. In contrast, the patterns resemble nearly point maxima concentrations commonly formed by dislocation creep i.e. crystal-plastic deformation, in orthorhombic phases such as olivine or pyroxene^1^. In addition, subgrain boundaries in orthorhombic phases are commonly formed perpendicular to the mineral’s long axis and foliation^41^. We thus infer that the observed CPO and substructures were inherited from an orthorhombic precursor phase, and were preserved during subsequent inversion to the lower-pressure cubic chromite structure^42^,^43^. The exsolution of diopside + SiO_2_ suggests transformation from the HPP, which has orthorhombic symmetry^11^,^12^,^13^. Chromite domains exhibiting diopside exsolution are smaller than the average grain size. The observed grain-size and orientation characteristics imply that a finer-grained orthorhombic HPP was deformed by dislocation creep, and thus could develop a noticeable CPO. Chromite shows subtle deformation features unrelated to late decompression fracturing (Figs 1d, S1), confirming crystal plastic deformation as the origin of the chromite CPO. The latter deformation characteristics, along with the presence of rims of chromite without exsolution, suggest that grain growth under static conditions (lack of significant differential stress) occurred in the upper mantle, i.e. in the low pressure chromite stability field, resulting in some recovery of internal deformation structures, growth-related substructures in the rim^31^,^44^, increase in chromite grain size, and the present equilibrated triple junctions and straight grain boundaries (Fig. 1a). The preserved significant chromite CPO is the result of a combination of dynamic recrystallization of the HPP chromite precursor and significant static recrystallization at lower pressure^45^.

The diopside needles exsolved in a topotaxial relationship with host chromite, as shown in previous studies^10^ (Figs 2d, S3). This relationship is also shown by clinopyroxene lamellae in garnet from Norway, formed during the breakdown of a majoritic-garnet precursor^46^. Diopside does not show any significant crystal bending, implying that the diopside needles are likely to have exsolved *after* the crystal-plastic deformation recorded by the chromite CPO (Fig. 2d).

The strong CPO of the olivine indicates deformation by dislocation creep, as crystal alignment during compaction and/or flow of partially molten systems produces a weaker CPO^47^. Grain-boundary sliding and diffusion creep would produce even weaker CPOs^48^,^49^. In the last decade, there has been significant progress in understanding the crystal-plastic deformation of olivine at high pressure, as well as that of wadsleyite in its stability field. Olivine CPO characterized by [001] axes aligned parallel to the shear direction (i.e. foliation) has been observed in experiments^50^,^51^ and predicted by numerical modelling of crystal plasticity^52^, and has been attributed to the presence of water and/or pressure. In this study, olivine shows the (010)[001] fabric, which we interpret to record the peak PT conditions. This was preserved during late exhumation due to a general lack of significant differential stress at lower P, as shown by the lack of significant deformation overprint on the inverted chromite, and entrapment in the rheologically stronger chromite host, shielding it from subsequent deformation. If later deformation changed CPO patterns, we would expected this to be more pronounced in interstitial olivine grains, as inclusions would be protected. However, the CPO patterns of inclusions and interstitial olivines are identical (Fig. 2b). The fact that inclusions with the characteristics shown here are only found in the exhumed UHP chromite, (identified by the exsolution of diopside and SiO_2_), further supports our interpretation that the olivine CPO was produced by deformation under UHP conditions.

Olivines hosted in chromite (both inclusions and interstitial grains) have a (010)[001] fabric, which we interpret as reflecting deformation by dislocation creep within the MTZ. This implies that these olivine grains were deformed and aligned while in one of the higher-P polymorph states. Wadsleyite (or β-olivine) is volumetrically the main component of the upper transition zone, between 410 and 520 km depths, and is not stable at shallower depths. Deformation experiments on wadsleyite show several active slip systems^53^,^54^,^55^,^56^,^57^; for example, (010)[100]^53^, (010)[001]^53^, {011}[100]^54^, (010)[100]^54^, (001)[100]^54^, (001)[010]^54^, (010)[100] (dry conditions)^55^, (010)[001] (dry conditions)^56^,^57^, and (100)[001] (wet conditions)^55^,^56^. The phase transformation from olivine (ol) to wadsleyite (wds) with topotaxial relations was studied at 13 Pa and 1400 °C using a multi-anvil apparatus^58^, and occurs as two types; (1) (101)_wds_//(100)_ol_ and [010]_wds_//[001]_ol_, (2) (021)_wds_//(100)_ol_, (011)_wds_//(010)_ol_ and [100]_wds_//[001]_ol_, respectively. Although these topotaxial relationships are the result of the phase transformation from olivine to wadsleyite, crystallographic considerations predict that they are expected also to be applicable to that from wadsleyite to olivine, because the close-packed oxygen layers of olivine and wadsleyite cause these topotaxial relations. Integration of our data indicates that the observed (010)[001] fabric preserves the (011)[100] fabric of wadsleyite despite the transformation. Such a fabric is most consistent with simple shear deformation of “dry” wadsleyite under MTZ conditions^54^.

## Conclusions

**Figure 3:**
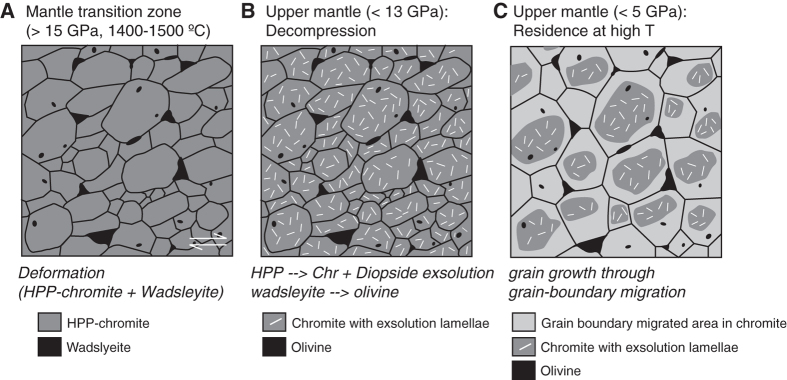
Schematic model of microstructural evolution in Luobusa chromitite. (**A**) Deformation of HPP (High Pressure Polymorph)-structured chromite and wadsleyite in mantle Transition Zone. (**B**) Transformation from HPP structure to chromite; diopside/coesite exsolution; transformation of wadsleyite to olivine. (**C**) Grain growth through static grain-boundary migration to produce coarse equilibrated microstructure, with exsolution-rich cores and exsolution-free rims. Larger interstitial olivine grains pin the migrating boundaries.

The significant CPO of included and interstitial olivine grains in chromitite implies the activation of the (011)[100] slip system in wadsleyite during crystal-plastic deformation by dislocation creep under MTZ P-T conditions. The transformation from an orthorhombic HPP-chromite to cubic chromite during exhumation drove the exsolution of diopside and coesite, and the wadsleyite grains transformed to olivine during this process, while preserving the wadsleyite CPO. Significant grain-boundary migration under static conditions during passive exhumation produced equilibrated microstructures but preserved the core-rim structure with exsolved diopside only in the cores (Fig. 3), as well as the high-PT fabric. The relict olivine-chromite fabric of this Luobusa chromitite provides the first direct evidence of significant crystal-plastic deformation in the Mantle Transition Zone.

## Methods

Chemical compositions of host chromite and olivine inclusions in the thin section were analyzed with Cameca SX-100 electron microprobe at Geochemical Analysis Unit (GAU) at the Australian Research Council Center (ARC) of Excellence for Core to Crust Fluid Systems (CCFS) in Macquarie University, Australia). Quantitative analyses were performed with an accelerating voltage of 15 kV, 12 nA beam current. Crystallographic orientation measurements were obtained using the SEM-EBSD facilities at GAU at the ARC Center of Excellence for CCFS (Macquarie University, Australia) and Australian Centre for Microscopy and Microanalysis (University of Sydney, Australia). The EBSD patterns were generated by the interaction of a vertical electron beam with a polished thin section, tilted at 70° to the horizontal in a scanning electron microscope (Zeiss EVO MA15 and Zeiss Ultra Plus). The operating conditions were a voltage of 20 kV, a current of 8.2 nA and working distance of 12–13 mm. The diffraction pattern was projected onto a phosphor screen and recorded using a digital CCD camera. The resulting image was then processed and indexed in terms of crystal orientation using the AZtec software distributed by Oxford Instruments. Maps were acquired with sampling step size of 20 μm, 1–5 μm or 200 nm. Raw indexation rates were >85%. Data treatment allowed the rare non-indexed pixels to be filled, if up to six identical neighbors existed with this orientation. We present the resulting data in the form of color-coded maps and pole figures. For the maps that show crystal orientation changes relative to the specific direction of the sample reference frame, full red, green, and blue colors are assigned to the grains whose <100>, <110> or <111> axes are parallel to the projection of the inverse pole figure. Intermediate orientations are colored as a mixture of the primary axes. For our analysis, we define (i) a grain as an area that is completely surrounded by boundaries with a misorientation of 10, and (ii) a grain size of equivalent circle diameter calculated by grain area. One point per grain data are plotted on pole figures using programs developed by D. Mainprice of Université Montpelier II, France.

## Additional Information

**How to cite this article**: Satsukawa, T. *et al.* Messengers from the deep: Fossil wadsleyite-chromite microstructures from the Mantle Transition Zone. *Sci. Rep.* **5**, 16484; doi: 10.1038/srep16484 (2015).

## Supplementary Material

Supplementary Information
