# Supplementary material for: Messengers from the deep: Fossil wadsleyite-chromite microstructures from the Mantle Transition Zone
Source: Sci Rep. 2015 Nov 13;5:16484. doi: 10.1038/srep16484 (PMC4643243; doi:10.1038/srep16484)
Supplement: Supplementary Information [file srep16484-s1.pdf]

Supplementary information

Messengers from the deep: Fossil wadsleyite-chromite microstructures from the Mantle Transition Zone

Takako Satsukawa[1]*, William L. Griffin[1], Sandra Piazolo[1] and Suzanne Y. O'Reilly[1]

[1] *Australian Research Council Centre of Excellence for Core to Crust Fluid Systems/GEMOC, Department of Earth and Planetary Sciences, Macquarie University, Sydney, NSW 2109, Australia*

Table S1. Average EMP major element composition of host chromites and olivine inclusions.

Mineral (Number of measurements)	Chromite Host (10)	Olivine Inclusions (44)
wt.%		
SiO_2	0.00	41.97
TiO_2	0.21	0.00
Al_2O_3	10.94	0.00
Cr_2O_3	61.56	0.00
FeO	11.81	1.87
MnO	0.15	0.00
MgO	15.87	55.54
NiO	0.19	1.27
CaO	0.00	0.00
Na_2O	0.00	0.00
Total	100.73	100.65
Mg#	70.5	98.2

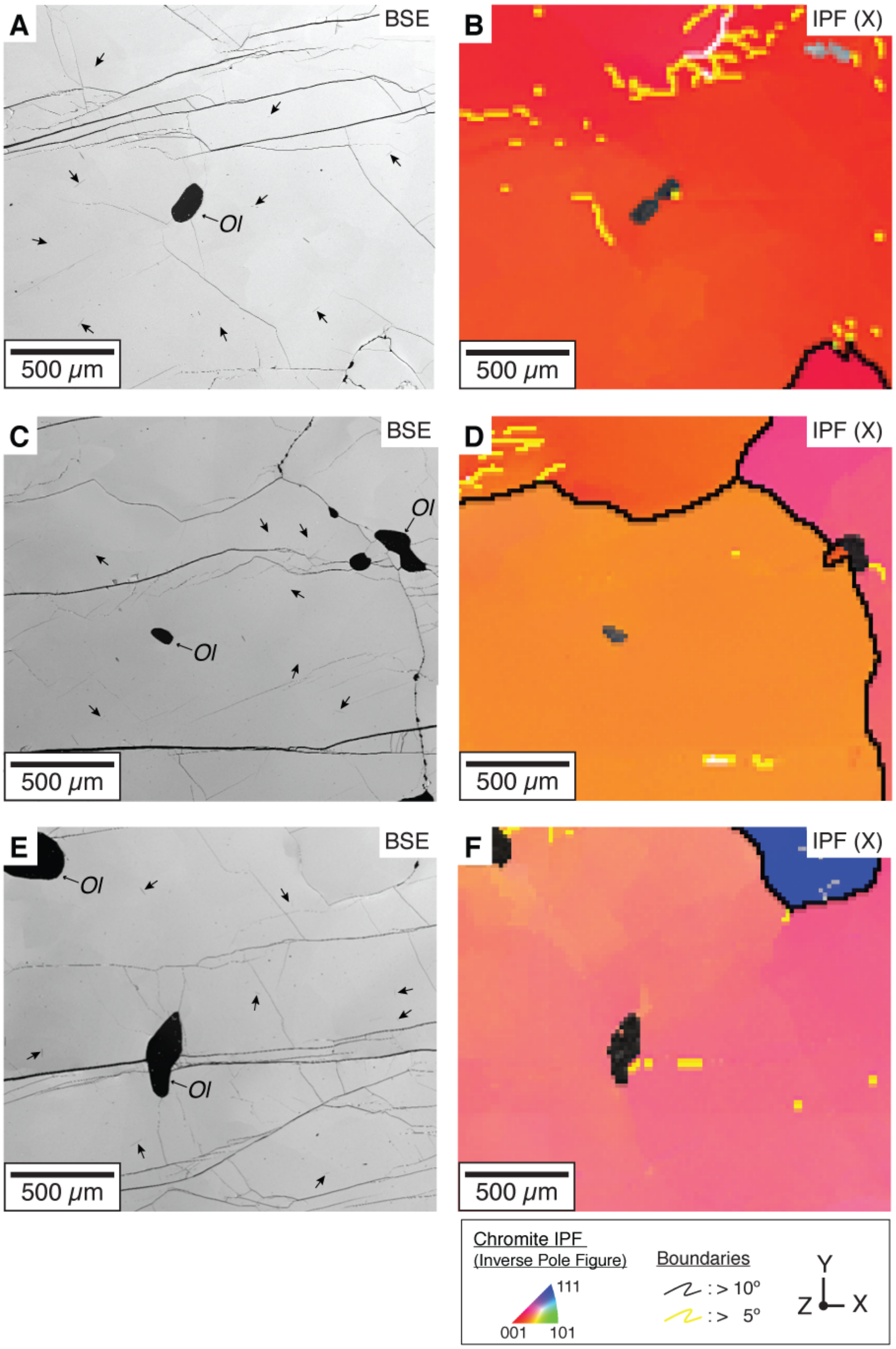

Figure S1. Olivine inclusions and surrounding exsolutions in the chromite grain. Close-up image of chromite in Figure 1(D). (**A, C, E**) Back-scattered electron image of chromite, which represent the exsolutions indicated by black arrows. (**B, D, F**) Colour coded EBSD map showing crystal orientation changes relative to the X direction of the sample reference frame. Black and yellow lines represent grain boundaries and subgrain boundaries, respectively. (**A, C, E**) and (**B, D, F**) represent the same area, respectively. Note: late fracturing related to decompression is clearly seen in BSE images. EBSD map shows some continuous internal deformation of chromite grains, some apparent subgrain boundaries are related to fractures.

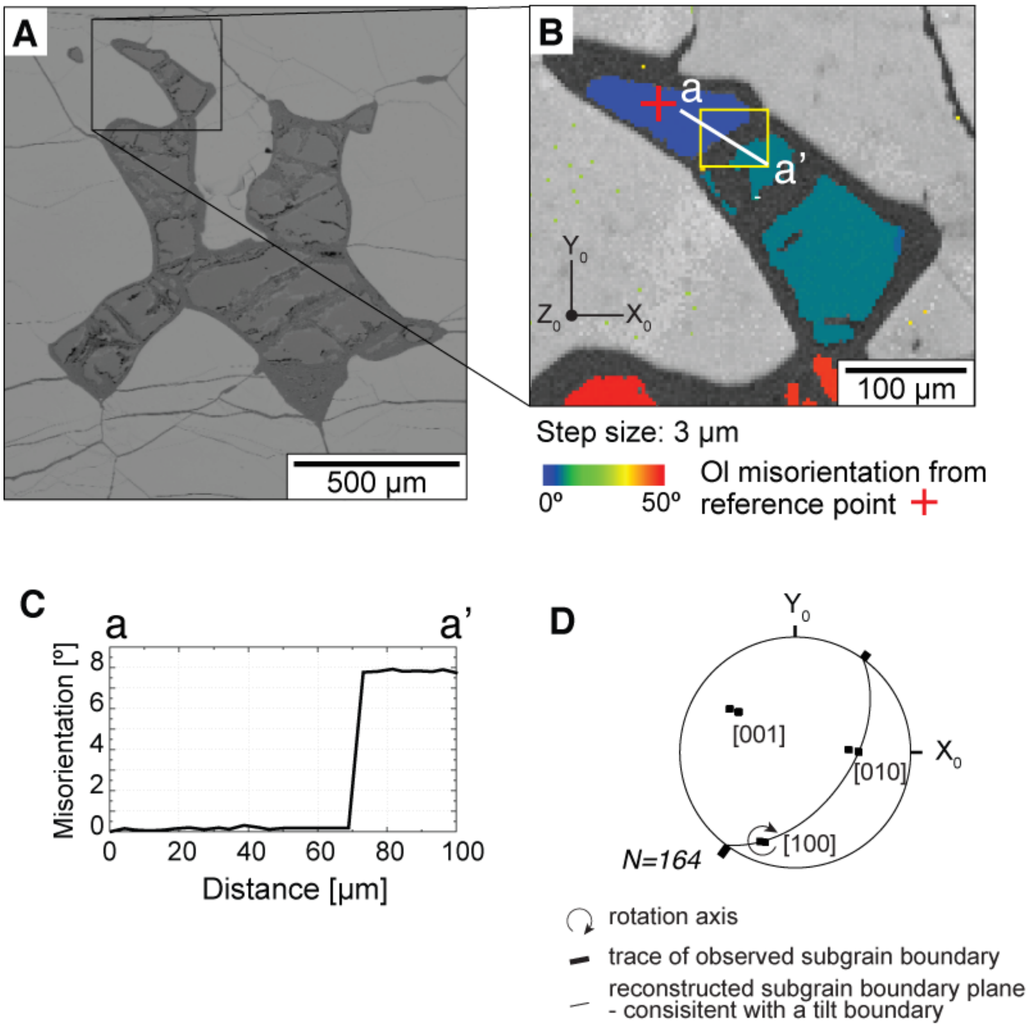

Figure S2. Subgrain-boundary analysis of two olivines. (**A**) Back-scattered electron image of interstitial olivines in chromite. They look like small grains, but the apparent small size is due to serpentinization. (**B**) Cumulative EBSD orientation map showing misorientation relative to crystal orientation at the position marked by the red cross. (**C**) Misorientation profile along the dotted white line in (**B**). (**D**) Olivine CPOs in EBSD map shows rotation axis is [100] and slip system is (010)[001].

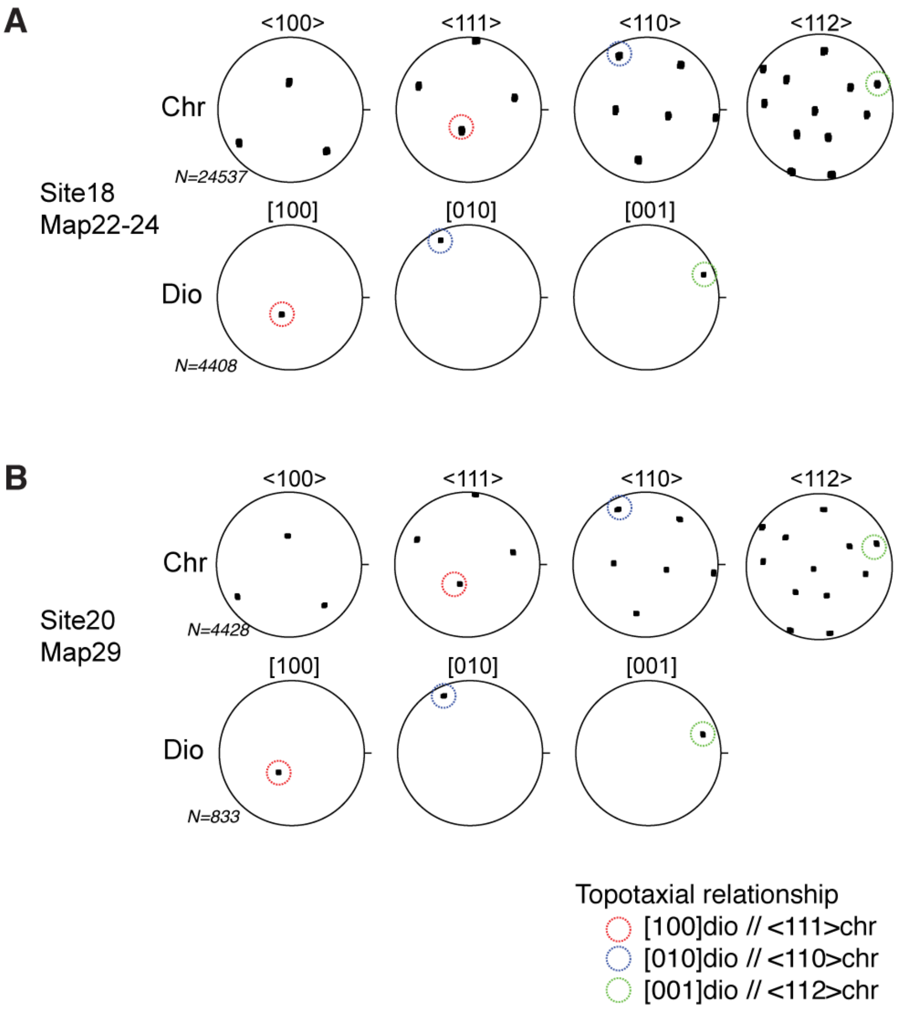

Figure S3. Orientation relationship of chromites (Chr) and included diopside (Dio) exsolutions. Lower hemisphere, equal-area stereographic projections. N is the number of measurements. Their orientation relationship are $[100]_{dio}$ // $<111>_{chr}$, $[010]_{dio}$ // $<110>_{chr}$, and $[001]_{dio}$ // $<112>_{chr}$, respectively.
